# Toward including environmental sustainability in Health Technology Assessment

**DOI:** 10.1017/S0266462325100500

**Published:** 2025-09-22

**Authors:** Anke-Peggy Holtorf, Melissa Pegg, Debjani Mueller, Nicola McMeekin, Environmental Sustainability in HTA Working Group of HTAi (ESHTA)

**Affiliations:** 1 Health Outcomes Strategies, Basel, Switzerland; 2 College of Pharmacy, University of Utah, Salt Lake City, UT, USA; 3 ESHTA at Health Technology Assessment International (HTAi), Edmonton, Alberta, Canada; 4 York Health Economics Consortium, University of York, York, UK; 5 University of Pretoria, Pretoria, South Africa; 6 Health Economics and Health Technology Assessment, School of Health and Wellbeing, University of Glasgow, Glasgow, Scotland

**Keywords:** environmental sustainability, Health Technology Assessment, HTA, value framework

## Abstract

**Introduction:**

The life cycle of health technologies contribute to air pollution, ecotoxicity, and resource depletion, impacting the environment and human health. Increasing healthcare resource use globally increases emissions that accelerate climate change and negatively affect the health of current and future generations.

Health Technology Assessment (HTA) should inform decision makers to prioritize the adoption of technologies demonstrating value in terms of health benefits, costs, and other relevant dimensions such as environmental sustainability.

This paper reports on a multistakeholder approach to guiding an international working group for Environmental Sustainability in Health Technology Assessment (ESHTA) that has been formed by Health Technology Assessment international.

**Methods:**

A multistakeholder online workshop was held with 32 participants in May 2024 to define the critical issues to be considered. The resulting report underwent consultation among the ESHTA members and in a broader group of 90 additional worldwide stakeholder representatives.

**Results:**

The workshop participants recognized defining frameworks, mechanisms, and tools for embedding environmental sustainability into HTA as an opportunity to support sustainable development and quality improvement in healthcare. Achieving this requires (1) consensus on what environmental sustainability in healthcare means, (2) reconcilement with other healthcare and environmental policies, and (3) methods that are useful and applicable within HTA frameworks.

**Conclusion:**

This novel collaboration aims to align the global HTA community on the role of environmental sustainability in HTA. The report provides a path for the way forward for incorporating environmental sustainability into HTA based on broad perspectives from global multistakeholders.

## Introduction

Healthcare contributes five percent of global greenhouse gas (GHG) emissions, with sixty-two percent of its carbon footprint attributable to the supply chain (1;2). The life cycle of health technologies contribute to air pollution, ecotoxicity, and finite resource depletion and affect the environment and human health (3). Chemicals and metabolites widely present in healthcare products include endocrine disruptors, carcinogens, mutagens, and substances that are toxic to reproduction (4). In addition, climate change is associated with the increasing prevalence of diabetes (5) and respiratory disorders (6).

Data on environmental harm attributed to pharmaceutical pollution are growing, including antimicrobial resistance, a leading global health threat (7). Since 2019, COVID-19-pandemic-related healthcare resource use has resulted in an estimated thirty-six percent increase in healthcare-related GHG emissions (2).

Nevertheless, despite being included in its definition (8), environmental aspects have not yet been broadly considered in Health Technology Assessment (HTA). HTA should inform decision makers to prioritize the adoption of technologies demonstrating value in terms of health benefits, costs, and other relevant dimensions. Considering the environmental consequences of the health technology (referred to herein as technology) lifecycle on planetary health and, hence, on the health of current and future populations, environmental sustainability aspects could be a critical modulator of the value (overall health benefits and societal costs) of a technology (9).

Moreover, environmental sustainability is a core pillar of sustainability defined by the United Nations as “meeting the needs of the present without compromising the ability of future generations to meet their own needs.” Hence, healthcare resources should be allocated efficiently to support current and future generations to meet their own health-related needs.

Therefore, a novel working group, “Environmental Sustainability in HTA” (ESHTA) (https://htai.org/engage-with-us/working-groups/htai-eshta/), was initiated within Health Technology Assessment international (www.htai.org, HTAi) to explore how environmental sustainability could be included in HTA.

In this study, we report on the initial line of enquiry developed by ESHTA through a multistakeholder workshop and subsequent wider consultation process.

## Methods

### Conceptualization workshop

A workshop was conducted in English language in May 2024 with thirty-two ESHTA members. It included seven HTA practitioners (England, Germany, Italy, Scotland, Spain, Taiwan, and Thailand), eighteen researchers (Belgium, England, India, Italy, Netherlands, Scotland, Spain, South Africa, Thailand), a public health expert (UK), and five employees of health technology developers (HTDs; UK and global companies). No clinician or patient participated in the workshop, but these stakeholders were included in subsequent consultation rounds.

A prestructured Miro whiteboard (https://miro.com/) allowed participants to contribute during the workshop. The board was organized by perspective (i.e., HTA agencies, health policy researchers, industry, patients, clinicians/providers, and public) to stimulate the participants to add themes. Phases of individual work followed by group discussion enhanced individual contributions while also allowing for interactive reflection. Two tasks were assigned to the participants:

- They were asked to formulate, from their own perspectives, their expectations or concerns for incorporating environmental sustainability into HTA.
- They were then asked to comment from other stakeholders’ perspectives or to comment on others’ contributions.

All ESHTA members (n = 50 in May 2024) had access to the Miro board for two weeks following the workshop, which enabled members who could not attend the workshop to add their views and comments.

### Processing of the workshop data

Data posted on the Miro board (121 posts) were transcribed into an Excel spreadsheet and then aggregated into five categories (Supplementary File 1).

- Objectives (ambitions of ESHTA): What would be the goal of integrating environmental sustainability into HTA? (20 posts)
- Tools/requirements: What requirements need to be fulfilled, or which methods are needed to integrate environmental sustainability into HTA? (22 posts)
- Processes/enablers: What enabling factors may be considered? (32 posts)
- Implementation measures: What facilitates the implementation? (32 posts)
- Posts that were out of scope or did not fit any category related to the goals of the workshop (15 posts).

The results were summarized in a draft report.

### Consultation process

Two rounds of consultation were conducted.

1. Consultation Among ESHTA WG Members.

A workshop draft report was sent to all ESHTA members in October 2024 (then n = 90) to provide feedback within two months. The consultation comments informed the report revision and this manuscript.

2. Wider Consultation. *HTA Researchers or Practitioners*

The revised report was shared with more than ninety additional stakeholders representing a broader range of perspectives. These were HTA practitioners/researchers, patient experts, government representatives, regulatory experts, climate change experts, and clinicians contacted through the ESHTA leaders’ networks. An accompanying email outlined the background and asked for critical reading and feedback within two months using a feedback template. The comments and feedback were collected and were considered in the discussion of this paper.

## Resulting reflections

### Objectives

HTA aims to facilitate health policies and decisions that foster equitable, efficient, and high-quality healthcare systems. Defining a framework, mechanisms, and tools for embedding environmental sustainability into HTA was perceived by the workshop participants as an opportunity to support, in addition, sustainable development and quality in healthcare.

A range of outcomes were formulated as long-term objectives of incorporating environmental sustainability into HTA:

- Achieve **societal acceptance** of environmental sustainability aspects as essential criteria – next to effectiveness and economic aspects – for setting healthcare priorities.
- **Policy makers and regulators** set **environmental targets and acceptability ranges** potentially supported with incentives (prioritization, preferred listing) or disincentives (limitation).
- **Physicians, pharmacists,** or other **healthcare professionals** consider environmental sustainability aspects in their **prescriptions** without compromising health outcomes. This could mean implementing strategies for technology-use optimization, waste reduction, or reduction in travel needs for patients.
- **Providers** (providing healthcare diagnosis and treatment services) adopt environmental sustainability aspects into their **local or organizational decisions**, for example through mitigating the need for healthcare interventions and adopting circular economy strategies with established and new technologies. This could include repair, reuse, or repurposing of such technologies at the end of their intended use.
- **Healthcare professionals** (individuals providing healthcare treatment and advice based on formal training and experience) are enabled to make more **sustainable healthcare decisions across care pathways**. This could be based on value assessments across technology life cycle stages, for example guidance or decision tools for healthcare professionals enabling the provision of nonpharmacological interventions with less environmental impact.
- **Patients** are empowered to make more **informed choices** between technologies based in part on products’ environmental credentials. For example, decision aids could help them understand the trade-offs related to different therapies.
- **Healthcare industries** have **clarity** on how and where to integrate **measures for environmental sustainability** into development programs and can submit their progress and investment in sustainability in the assessment.

### Tools

Creating a framework for evaluating and incorporating environmental sustainability into HTA requires consensus on:

1. the definition of environmental sustainability in healthcare,
2. its relevance and alignment with healthcare policies, the remit of HTA, or other institutions, and
3. the methods and their usefulness and feasibility within the HTA framework.

Herein, it will be instrumental to identify the appropriate assessment parameters for environmental sustainability and to understand the acceptability or risk ranges for each of the assessment parameters. This could include estimating the impact on human health, natural systems (planetary health, including water and ocean health), and resources required for using technologies. Understanding the relative contributions of different technologies to environmental sustainability aspects will help prioritize technologies in healthcare decision making.

**Figure 1.**
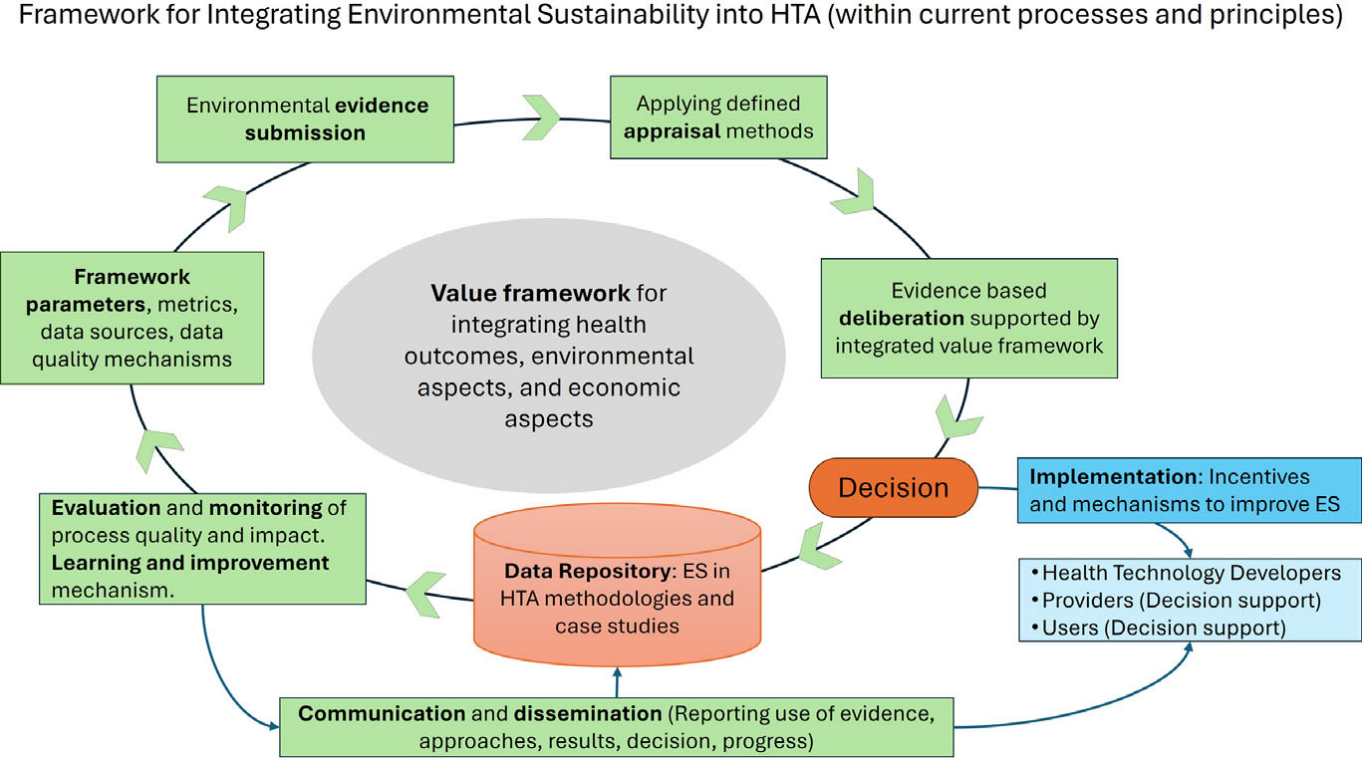
Framework and methodological approaches for integrating environmental sustainability (ES) into HTA (within current processes and principles).

The framework structure and methods should encompass the entire HTA pathway, including early HTA, evidence submission, analysis, appraisal, value-based healthcare prioritization, and recommendations, as shown in Figure 1. However, a fully value-based approach that integrates environmental sustainability (10) may introduce methodological complexities and challenges that cannot yet be overcome. Information gaps currently limit continuous “living HTA” concepts (11). Parts of the technology life cycle may be beyond the ability (resources) and remit of HTA and fall into the responsibility of others (to be defined). Therefore, feasible and pragmatic approaches to including environmental sustainability in HTA are required. This may mean focusing initially on areas, processes, or activities with potentially more significant environmental impact (“environmental hotspot” analysis).

Incorporating an environmental sustainability framework into HTA requires – potentially next to research by the HTA agency – the appraisal of environmental data and evidence submitted to the HTA agency. For this, stakeholder groups should agree on the approach and format for submission, the type of data, acceptable data sources, quality criteria, and validation methods.

**Figure 2.**
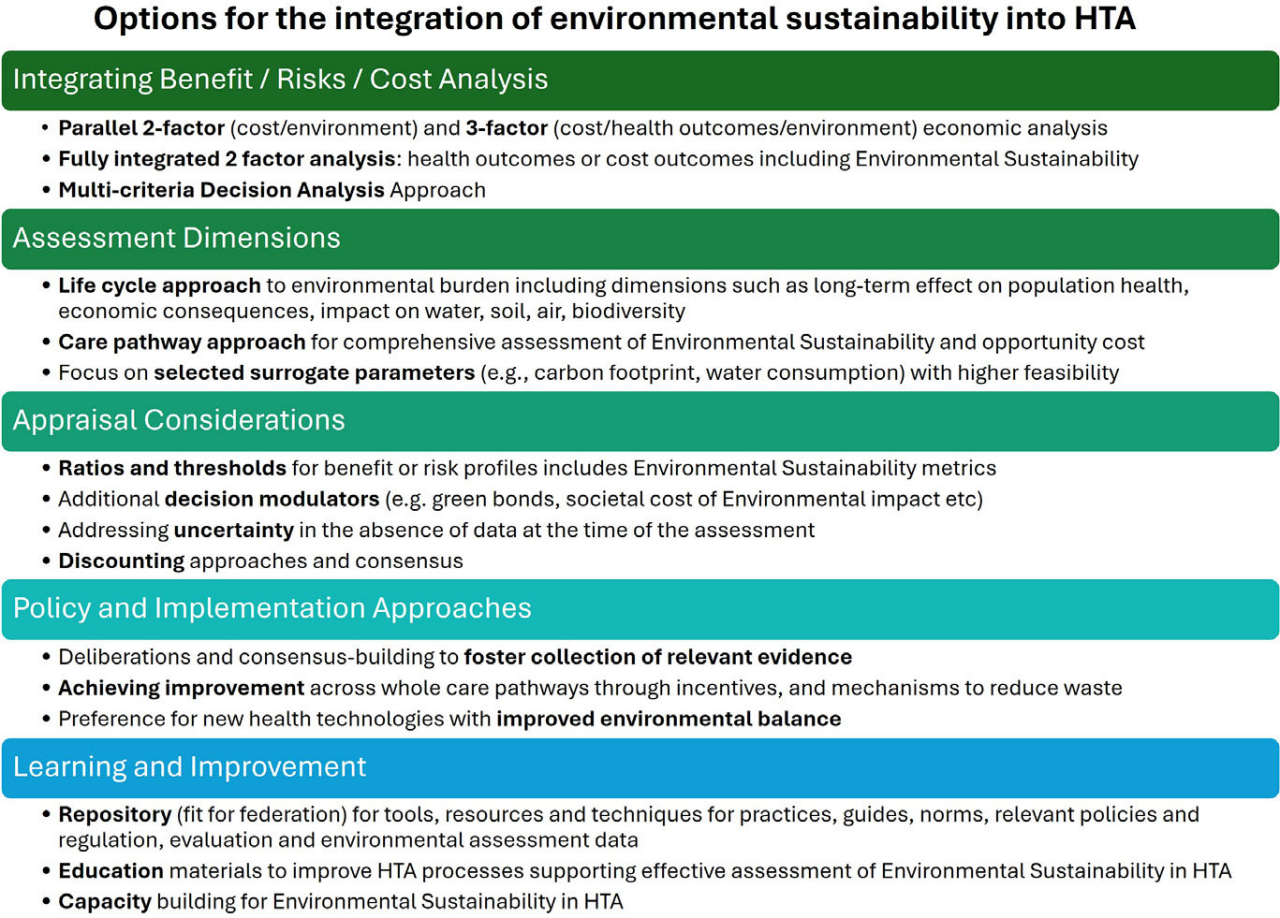
Important aspects and options for the integration of environmental sustainability into HTA.

Consensus needs to be built on several options (Figure 2) before the assessment of environmental sustainability can be fully integrated into HTA. Appraisal methods and potential use of analytical tools need to be defined, including ratios and thresholds for a benefit–risk profile, accounting for additional decision modulators (e.g., green bonds as fixed-income investments in projects with positive environmental impact or debt-for-nature swap mechanisms whereby a country’s debt is reduced in return for committing a proportion of the reduction for environmental protection), and uncertainty in the absence of data at the time of HTA.

Furthermore, legislation, healthcare and policy priorities, HTA reference manuals, and value frameworks can shape decision making within and across jurisdictions. Instead of static decisions (e.g., to reimburse or not, price accepted or to be negotiated, limitation of use due to environmental credentials), a dynamic range of recommendations might be considered to facilitate the implementation of the decision, as shown in the lower right corner of Figure 1. Outcome-based targets could be defined to guide further life cycle decisions related to each technology (e.g., incentivization of preventive public health interventions, resource optimization across care pathways, and reprioritization). Therefore, the assessment itself could directly contribute to improvements adopted by HTDs and users.

To accelerate change, methods will have to be refined and improved while already in use. Hence, alongside data collection (data repository) and evaluation aiming to inform learning and improvement, it is proposed to ensure transparent communication and foster a collaborative approach and dialogue between the stakeholders concerned (e.g., policy makers, HTA, industry, economists, patients, and clinicians).

### Processes: how to get there?

**Figure 3.**
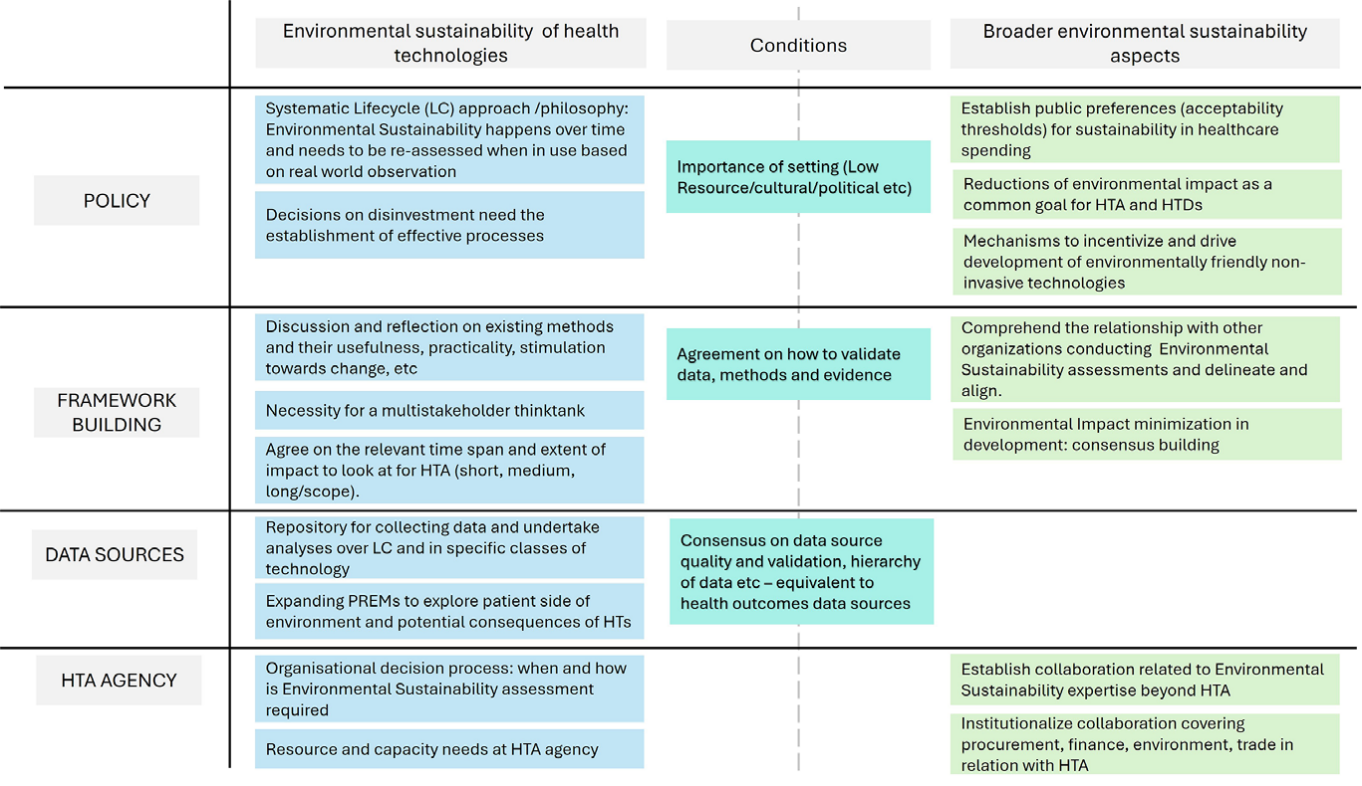
Steps for moving toward more systematic consideration of environmental sustainability in HTA. This summary is based on the workshop data outlined in Supplementary File 1 (How do we get there?). HTDs, health technology developers; LC, life cycle; PREMS, patient-reported outcome measures.

Figure 3 summarizes the proposed steps toward more systematic consideration of environmental sustainability in HTA.

*Policy levers.* To build environmental sustainability into the remit of HTA agencies, supporting policies are needed as well as consensus on suitable frameworks and environmental metrics. Furthermore, the environmental footprint of care may change over time; therefore, adaptation and re-evaluation of environmental sustainability is a continuous task in HTA, rather than a one-time event. Policy infrastructure may eventually allow for building a product environmental life cycle database and its environmental impact along the care pathway. Therefore, accumulation of knowledge over time – complemented with appropriate feedback and improvement mechanisms – could enable learning and adaptability.

Environment-conscious HTA should also inform value-based (health, cost, and environmental outcomes) purchasing or procurement of technologies. Where trade-offs between environmental sustainability and impacts on patient benefit, social, or financial factors are necessary, careful consideration and policy guidance will be required. Understanding jurisdiction priorities and the local–national context (healthcare system, political system, economic situation, cultural beliefs, etc.), as well as associated dependencies and challenges, will help overcome related barriers in the implementation phase.

Any standards for integrating environmental sustainability into HTA need to match the goals and principles of HTA. Multidisciplinary action between policy makers, HTDs, and other key stakeholders involved in healthcare decision making – aligned with broader government-led sustainability targets – is crucial (12). Consequently, incentives (e.g., preferred access) and disincentives (e.g., limited access, pricing adjustments, or value-based prioritization) may be introduced depending on jurisdictional priorities (13).

*A Value Framework.* An environmental sustainability framework should build on values, methods, and quality standards informed by multistakeholder consensus. Therefore, ESHTA recommends inclusive stakeholder collaboration and cocreation to establish a value framework that can be implemented at pace. Key stakeholders are health policy and HTA experts, regulatory bodies, environmental experts, patient experts, healthcare professionals, researchers, consumer representatives, providers, payers, HTDs, and others with a strong interest in healthcare and environmental sustainability. Broad-spectrum agreement and consensus among stakeholders could facilitate effective implementation and scalability.

*Data Management.* Standards for evaluating environmental sustainability and incorporating it into value assessment and decision making do not yet exist. Given the scarcity of environmental impact data and related uncertainty, data handling is critical. Environmental management standards, guidance, and frameworks from other sectors could help overcome the challenges in prioritization, risk stratification, and environmental data management. Early HTA could advise HTDs to collect appropriate data that support such assessments. Furthermore, coordinated data access and exchange across relevant stakeholders could support the accordance between regulators, public-sector policy makers, and buyers.

*Perspectives at the HTA agency level.* Resources are needed for the assessment of the environmental sustainability dimension that will likely call for appropriately skilled personnel and organizational adaptations. HTA agency infrastructures need to cater to the integration of environmental sustainability methods across the entire HTA spectrum.

Interfaces with other organizations and disciplines with expertise in environmental sustainability and environmental management need to be established (e.g., environmental government institutes, technical surveillance institutions, or hospital management). Building transdisciplinary collaborations and synergies with other departments (in the hospital) or policy sectors (on the jurisdictional level) could help improve efficiency. As a part of this, clear delineation of responsibilities is important to avoid duplication and redundancy. For example, Hensher suggests that HTA should focus on technology-intrinsic aspects, whereas others (e.g., regulatory or technical surveillance agencies) should control generic environmental measures (14).

### Implementation: how will change happen?

Progress requires learning for HTDs, HTA agencies, and other stakeholder groups, which could be prioritized according to environmental risks or known environmental hotspots in healthcare. A key contribution could be collaborative pilot HTAs that test how to include environmental outcomes and analytical techniques and how to consider the outcomes within decision-making frameworks. Herein, opportunity cost, relative trade-offs, and related value frameworks need to be understood. Broader adoption requires technical guidance (using, for example, reference cases) depicting validated methodologies for assessing environmental sustainability. Procedures are required for method integration into existing HTA processes, including the integration of established multisectoral frameworks, such as One Health (e.g., to evaluate pharmaceuticals) and circular economy (e.g., to evaluate medical devices/equipment). Methods and guidance should be developed in communication with core stakeholders, governments, and HTDs, to create techniques that are feasible, effective, and broadly accepted.

To support a felicitous response to establishing environmental sustainability in HTA, substantiating legislation could include:

1. supporting environmental data generation and submission by developers,
2. environmental monitoring of chemical metabolites and novel entities of health technologies, including pharmaceuticals, biologics, medical devices, or public health interventions,
3. reporting of environmental outcomes measures, including mitigation and adaptation measures within clinical trials and throughout the HTA continuum, and
4. the inclusion of environmental impact dimensions in guidance for the use of health technologies.

Legislation or guidance could be established on an international level such as through the Organization for Economic Co-operation and Development (OECD) and the United Nations (UN). Promotion is needed to increase general awareness about the environmental aspects of healthcare. Incentives for minimizing the environmental impacts of health technologies or increasing preventive healthcare should complement and facilitate top-down legal frameworks.

Patients are value holders. Self-care is a key factor in sustainable healthcare, helping people to take more control of their health, which can lead to better outcomes and more efficient healthcare systems. Alongside value holders themselves, healthcare professionals are key influencers. Where environmental sustainability collides with patient preferences and suggests limiting patient choice and access to environmentally more harmful technologies, careful communication is required.

On the consumer side, implementation of environment-conscious decision processes will also require consensus, education, and decision support tools for healthcare providers, clinicians, and the general population. Subsequently, environmental sustainability aspects in HTA should be translated into healthcare delivery. For example, guidance to support resource optimization and effective use of health technologies will help counteract undesirable environmental, financial, and social impacts of health technologies. Case studies, such as strategies to mitigate antimicrobial resistance, could be useful and help test and refine value frameworks.

In summary, closing the implementation loop for achieving sustainable healthcare requires the inclusion of legislation, environmental, financial, and social sustainability factors, education, communication, collaboration, and support tools that facilitate integrated, inclusive, and sustainable adoption.

## Discussion

There is no widespread consensus yet on the role of environmental sustainability in HTA. Different – sometimes controversial – priorities and a lack of common direction for achieving environmental benefit across policy, regulatory, procurement, and clinical stakeholders may create significant challenges for developers and suppliers of health technologies. Therefore, HTAi initiated ESHTA to explore the state of the art and outline a path forward. An initial workshop was held by ESHTA to frame its objectives and work. The workshop deliberations yielded short- and long-term objectives that may also help inform the work of other initiatives for promoting environmental sustainability in HTA.

The current and projected state of planetary health exacerbates the burden on global healthcare systems and resources. Globally, limited healthcare budgets are stressed further by growing demand for services attributed to environmental breakdown (9). This worsening situation adds to healthcare system instability, escalating pressure on and vulnerability of population health, and increasing the need for healthcare resources (15).

Specifically, lower-resource settings (LRSs) are generally experiencing greater and more frequent climate change effects than higher-income settings (16). It is proposed that LRSs will benefit from greater commitment to environmental sustainability in healthcare – being a common responsibility of all countries consuming resources and thereby contributing to environmental effects. Evidence-based practices adapted to local contexts, value-based assessment of health technologies, and community engagement can support appropriate allocation of scarce resources and enhance decision making in LRSs.

In the workshop, several methodological approaches and concepts were proposed to evaluate a broad range of environmental impacts, including carbon hotspot analysis or the calculation of carbon emissions or other indicators along the product lifecycle and patient care pathway. Several environment-related initiatives were recognized as meaningful and potentially effective in contributing to healthcare decision making. Efforts should align with the principles of “sustainable quality improvement” (SusQI) (17;18), suggesting that “health outcomes of a service are measured against its environmental, social and economic costs and impacts to determine its overall value.” Importantly, although there is a need for a robust value framework to guide comprehensive and thorough assessments, there is also recognition that such a value framework should be pragmatic and “living” (adaptable based on experience, need, and evidence availability or accessibility) so as not to defer its use, particularly in the early days of implementation.

Improving the environmental sustainability of health technologies and healthcare requires commitment from government leadership globally. Prioritization techniques currently used by HTA organizations may allow the adoption of environmental policy targets and speed up the integration of environmental sustainability into HTA. Examples include environmental horizon scanning techniques, decision aids informed by environmental data, guidance that supports optimizing the use of healthcare resources in line with SusQI, trans-/multisectoral collaboration, and alignment with procurement frameworks. Therefore, environmental sustainability guidance and tools could incentivize new and ongoing initiatives in industry. Recently, ESHTA provided dialogue on how incorporating environmental sustainability into early HTA can enhance the likelihood of regulatory approval and reimbursement, ultimately benefiting patients and healthcare systems (19).

Crucially, extensive progress can be achieved where patient benefit accompanies reduced financial and environmental costs. However, conflicts may arise, where patient access would be weighed against population health of future generations and related transgenerational responsibility. Discourse and collaboration among the different stakeholder groups – including patients and the public in general – will be essential not only to be representative, but also to achieve consensus on change acceptable to society. This could include defining acceptability thresholds or other ways to support decisions related to the opportunity costs inherent in choosing among optimizing planetary, individual, and population health, as exemplified by Pegg et al. (20).

A cautious approach is also advised to assess and implement emerging, potentially disruptive interventions such as telemedicine or artificial intelligence. Herein, differences in the intrinsic characteristics of technologies, the demand for a technology depending on its use and indication, and consideration of healthcare contexts (e.g., low-, middle-, or high-resource settings) will warrant a case-by-case analysis of impacts on outcomes in health, environment, and equity.

To initiate environmental assessment despite HTA resource constraints, prioritization could follow a criteria-based risk classification system related to environmental sustainability (21) or classification by inherent or generic environmental aspects (14). Finally, to improve global stakeholder learning, a living, centralized, international repository for environmental sustainability data accessible for HTA across jurisdictions as exemplified by the work initiated in the HealthcareLCA database (22) could also be beneficial.

### Observations from the broader consultation of the report

The feedback received from the broader consultation of the report with stakeholders who had not been previously involved in ESHTA was generally very supportive. It included the observation that the most effective solutions may not be in adding technology but in doing less and incentivizing healthier and less consuming population behaviors.

Several patient experts emphasized the importance of involving patients throughout this work and committing to explicit terms of involvement. In fact, this should be done for any stakeholder group involved – including providers and clinicians – and should consider management of potential conflicts of interest through transparent declarations. Specifically, it was suggested that collaboration with clinicians could accelerate environmental sustainability gains in healthcare by scrutinizing current practices for wasteful activities or by integrating Circular economy across clinical decision-making processes.

Introducing environmental sustainability into HTA may increase the complexity and cost of HTA and hence hinder the rapid adoption of new technologies or even compromise access to existing care. For example, most healthcare systems currently rely heavily on the use of generic medicines where active pharmaceutical ingredients are produced in low-cost manufacturing environments that have been optimized for production efficiency, not for environmental sustainability. Hence, introducing new environmental standards could increase acquisition costs or exacerbate drug shortages. Likewise, development and production costs for new products may increase due to the need to fulfill the additional environmental sustainability criteria. However, this argument is true for other desired changes in the supply system such as improving product quality, working conditions, or production safety. Therefore, achieving such improvement will require a broader consensus in the broader health policy community.

Other feedback noted the need to view the planet Earth as “customer” and not only healthcare. In addition to developing processes and methods for integrating environmental sustainability in HTA, implementation requires educational programs for HTA researchers and across stakeholders involved throughout the HTA pathway.

There was also a suggestion to consider nominal group techniques for gaining consensus among a range of stakeholders as a more robust method than the one used in this initial workshop, which was designed to collect broad views of the participants.

### Limitations

The content presented in this paper resulted from preliminary discussions and a workshop with participants interested in this subject. Some stakeholder groups were not represented in these discussions, and clinicians and patients from different resource settings should be involved in the future, either directly or through consultation. The ideas, processes, and concepts presented here can only be the beginning of a longer and broader discourse and will have to be refined continuously to move toward meaningful and accepted propositions and solutions.

## Conclusion

The urgency to address climate change parallels the widespread recognition of the need for sustainable development in healthcare. Integrating environmental sustainability into HTA as an additional dimension may facilitate both. This inaugural ESHTA report provides broad perspectives from global multistakeholders along various touch points of the HTA process. This collaboration aims to frame the objectives, tools, and processes required to advance this field. We propose that the HTA community align globally and collaborate with other institutions on promoting environmentally sustainable technologies and services, aiming to improve the health of the current and future generations. Many questions are still to be answered. This report and the resulting workstreams of ESHTA may help pave the way forward.

## Supporting information

Holtorf et al. supplementary material Holtorf et al. supplementary material

## Supplementary material

The supplementary material for this article can be found at http://doi.org/10.1017/S0266462325100500.
