# Supplementary material for: Toward including environmental sustainability in Health Technology Assessment
Source: Int J Technol Assess Health Care. 2025 Sep 22;41(1):e70. doi: 10.1017/S0266462325100500 (PMC12516604; doi:10.1017/S0266462325100500)
Supplement: Holtorf et al. supplementary material [file S0266462325100500sup001.pdf]

Patients

Give a good decision base to choose between technologies if there are differences in term of EI					
Decision aids to support patient empowerment	Universal health coverage can only be achieved through sustainable healthcare systems that include decision making based on equal consideration of financial, social and environmental outcomes	Telemedicine			
Improvement in disposal and wastage of medicines					
More attention to PREMS also in respect to environment and potential consequences of health technologies on it.	Understand the balance (if any) between benefit risk profile and EI	Focus on quality improvement solutions - patient care pathways in the prevention space	Medicines optimisation	Patient centered care	
Clearly understand the timeframe involved by EIA (short, medium, long)					
Advise people living with specific disease on how climate changes will impact them and how they can cope with it.<br>Comment:<br>I was recently alerted to this aspect in a discussion with another group: there are two aspects to consider: (1) the impact health technologies on the environment, and (2) the impact of the changing environment on the people living with diseases. Examples: increasing heat during summer or more extreme weather, change of flora with impact on air composition etc.<br>I copied in an image relating to this from the World Economic Forum (APH)<br>- https://climos-project.eu/ (JM)					

HTA Agencies

Drive disinvestment in low-value care that imposes environmental costs (21)					
Clarification of whether and which environmental costs/outcomes can be accommodated within a health sector evaluation perspective					
Disinvestment from highly polluting HTs where alternatives are available (15)	Developing methods for 2-factor (cost/ environment) and eventually 3-factor (cost-health-environment) economic evaluations (15)	Promote/drive the development of health technologies with less EI (15)			
..and (following on from above define WHICH outcomes are of interest/importance to decision-making (10)	Integrated analysis is necessary to enable opportuntiy cost considerations within a health economic analysis				
Establish a think tank for discussion with different stakeholders					
Keeping patients first	Normative. Repository of experiences and methods	Disinvestment from highly polluting HTs where alternative are available	Apply circular economy research to MD HTA/hospital HTA/early HTA	More HTA focus on non-pharmacological interventions	
Validation of methodology and consensus building	Green HTA (EIA +HTA)	Need to focus on resource settings to achieve sustainability	Priority on social sustainability		
Guidances and methods					
LC approach					
Advise people living with specific diseases on how climate change will impact them and how they can cope with it	Much greater collaboration between procurement, finance and HTA agencies	Evidence based deliberation process and consensus building in ES in HTA to framework development	Scaling actions and create a PF on ES	lobyism for the adoption of those methods	Promote discussion on existing methods

Researcher/Academics

Dimensions to be evaluated: long term effect on population health, economic consequences, impact on other areas (water, soil, air)							
Database for LCA in healthcare - similar to costing database for EE							
Development of integrated framework between HTA and LCA methods							
Opportunity cost of investment							
Determine public preferences for sustainability in healthcare spending	Methodology: guidance/uniformity on what is/should be measured	eco-friendly health interventions	measure impacts on biodiversity. Do an LCA and<br>recyclability assessment				
minimum data							
LC approach, please no carbon footprint and economic impact	Provide guidance on evaluation methods. Help to design processes for integration of guidance	Develop standardized methods	Together with all stakeholders - to have something feasible and useful	Identify robust data sources to represent sustainability factors in economic models			
Development of new methods	Making decision sustainable from environmental point of view	Establish means of validating and appraising environmental information included as part of HTA submissions.	Ensure integration of methods in to process is efficient and meeting decision makers objectives	integrate in decision making			
**Industry**							
Prioritize patient outcomes - better outcomes, greener patients							
validation of methodology and consensus building							
categorization of intervention (by type/their EI)	link to early HTA<br>Comment: Just mentioning: This aspect is still uncertain, since the EIA is more likely to come into picture when a technology is commissioned and manufacturing & distribution best practices have been set in place by the HTD, however I feel an early HTA dossier should focus more on summarizing the issues that may arise based on experience. (ADM)						
LCA	Develop a position on what is needed from industry to define the methods	Use existing framework and not reinvent too much of the wheel	Consider sustainability outcomes in evidence development	Developing methods for 2-factor (cost-environmental) and eventually 3-factor (cost-health-environmental) economic evaluation			
Technical guidance contributing to incorporation/mainstreaming of environmental sustainability considerations into HTA and health benefit package design/revision	Understand data requirements						
Putting patients and disease management as first priority<br>Comment: Keeping people healthy and mitigating the need for intervention through disease prevention should be the first priority (MP)	Pragmatic approach and keeping uncertainty low	Provide clarity	Develop well accepted integrated (environmental (ALL ASPECTS) health, economic) sustainability method	industry consensus on what is and is not possible to provide in terms of data that could be used for assessments	Using a GRI as a basis	More HTA focus on non-pharmacological interventions	Opportunity cost of investement
**Public**							
Include endocrine disruption<br>Comment: toxicological impact (humans and animals) (APH)<br>This is a crucial part of the pollution consideration for pharmaceuticals and will link into green bonds starting to become part of the financial investment community considerations and in due course/environmental management of pharmaceuticals, for example (MP)							

Communication about decisions and underlying trade-offs that are being made including Environmental impact	link to early HTA						
Education on how healthcare is funded and the need to make more sustainable decision making based on social, financial and environmental outcomes	Create a dialogues between industry, HTA bodies, decision makers and citizens						
Develop standardized methods to formally include the EI in HTA	Understand the link between benefit-risk profile of a treatment and EIA<br>Comment: This is necessary as a framework and a "chapter" for environmental sustainability in HTA. AND to recognise that not ALL of the data points will be available for the LCA but that what is available and COMPARABLE will be included in the assessment (MP)<br>@Melissa Pegg agree absolutely with you! and we should try to capture and summarize the issues on why the data points are not available in different setting (ADM)	validated guidelines	Communication about decisions and underlying trade-offs that are being made including environmental impact	Evidence based deliberation process and consensus building in ES in HTA to framework development	Promote/ drive the development of health technologies with less environmental impact	Understand the impact of the healthcare sector as a whole and also of different diseases- looking at the benefits of prevention as well	Uptake of guidance by governments and industry
Potential integration with future value-based procurement methodologies (a strategic priority for NHSE) to reflect sustainability alongside other impacts (lifetime costs, operational efficiencies, improved patient outcomes)	Having more information of the environmental impact of healthcare	a new domain in HTA guidelines	Sustainable strategies	Technological innovation	Greater visibility of life cycle 'hotspots' by medtech category type (inform future healthcare strategy / policy)		
**Healthcare providers**							
Discuss sustainability with patients when discussing treatment options and allow for patient preference	Green procurement	Guidance to support AMR mitigation and adaptation of treatment pathways					
More involvement in HTA decision making to support more sustainable HTA	Integrating physician perspective	LMIC perspective:EIA vs unmet needs vs access	Consider ways to reduce medicines wastage across the system e.g. use of dates, storage	Developing a breathing process to include environmental impacts that has a balance between feasibility and the best available science- so basically developing standards that are continuously revised for inclusion of LCA and sustainability indicators.			
Provision of easily accessible ways of disposing of prescription medicines	Much greater collaboration between HTA agencies and hc providers/finance/procurement	Environmental calculations must include patient pathway. You can obliterate the carbon footprint of one product with an hour of surgery, an extra overnight stay, a re-operation or a shorter life battery	Clarify what we mean with environmental sustainability and why it's more thatn just legal requirements				
**Policy makers**							
We need legislation that requires environmental monitoring of chemical metabolites from health technologies including pharmaceutical and medical devices.<br><br>How do we know what to measure, assess and report if we dont know what we are looking for?	We need legislation that supports necessary reporting of environmental impact assessment criteria (mitigation and adaptation measures) within clinical trials and along the continuum of HTA (i.e. guidance, decision aids, decommissioning)	We require legislation that supports reporting a broader range of environmental outcomes than just carbon emissions.	We need legislation to support more sustainable HTA decision making				
**Others**							
Who is responsible for the assessment? HTA agencies or not only?	We need regulation to support environmental data generatoin from industry						

New resource needs/ expertise in ES	One Health framework/policies for pharmaceuticals	Clarify what we mean: do we asess the EI of the use and/or of the production of a technology? It's a multidimentional concepts to investigate	We need legislation to support more sustainable HTA decision making
Process to clarify: when the asssessmet is needed and how to montor it	Govt appropriate funding of preventative and non-pharmacological interventions		
Stakeholder engagement			
Democratise and open up sustainability assessment in the healthcare sector			
